# Impact of Anti-Citrullinated Protein Antibodies on Progressive Systemic Bone Mineral Density Loss in Patients With Early Rheumatoid Arthritis After Two Years of Treat-to-Target

**DOI:** 10.3389/fimmu.2021.701922

**Published:** 2021-06-14

**Authors:** Serena Bugatti, Laura Bogliolo, Antonio Manzo, Ludovico De Stefano, Paolo Delvino, Francesca Motta, Carlomaurizio Montecucco

**Affiliations:** ^1^ Division of Rheumatology, IRCCS Policlinico San Matteo Foundation, Pavia, Italy; ^2^ Department of Internal Medicine and Therapeutics, University of Pavia, Pavia, Italy

**Keywords:** rheumatoid arthritis, anti-citrullinated protein autoantibodies, bone mineral density, osteoporosis 3, rheumatoid factor

## Abstract

**Objectives:**

To investigate the association of anti-citrullinated protein antibodies (ACPA) with changes in systemic bone mineral density (BMD) in patients with early rheumatoid arthritis (RA) after two years of treat-to-target.

**Methods:**

BMD was measured at the lumbar spine (LS) and femoral neck (FN) in 100 patients with recent onset RA at baseline and after 24 months of treatment aimed at low disease activity (LDA) according to the 28-joints disease activity score (DAS28 <3.2). Multivariable regression analyses were performed to determine independent associations between autoantibodies and other disease and treatment-related parameters with BMD loss.

**Results:**

After 24 months, the majority of the patients were at least in LDA (78%), with slightly more ACPA-positive subjects achieving the target. The BMD had significantly decreased at both the LS (mean [SD] percent loss -1.8 [6.2], p=0.03) and the FN (-2.4 [7.3], p=0.03) in ACPA-positive but not in ACPA-negative patients. Consequently, the proportion of patients with reduced BMD (Z score ≤-1) after 24 months was significantly higher among ACPA-positive patients at both the spine (39.5% *vs* 19.3%, p=0.05) and the hip (37.2% *vs* 12.2%, p=0.007). The association between ACPA and BMD loss was independent of other variables including age, gender, disease activity, cumulative dose of glucocorticoids and duration of therapy with bisphosphonates at the LS but not the FN.

**Conclusions:**

ACPA are associated with ongoing BMD loss at the spine despite suppression of inflammation and adoption of prophylactic measures. ACPA-positive RA patients should be therefore strictly monitored for the development of osteoporosis.

## Introduction

Rheumatoid arthritis (RA) is a chronic immune-inflammatory disease characterized by destructive synovitis and pathologic bone remodeling, ranging from localized joint erosions to systemic osteoporosis (OP) (1, 2). Chronic inflammation is the major risk factor for progressive bone damage in RA (3, 4). However, several lines of experimental and clinical evidence have now consistently established the key contribution of autoantibodies in osteoclast-induced bone loss (5–7). In particular, anti-citrullinated protein autoantibodies (ACPA) are capable of promoting pro-inflammatory cytokine production and directly inducing osteoclastogenesis through the cross-linking of Fcγ-receptors as well as through the binding to citrullinated proteins expressed on the surface of immune cells and osteoclasts (8–10). Accordingly, ACPA-positive RA patients exhibit higher degrees of local and systemic bone damage (11–14) as well as reduced bone strength (15), and ACPA-positive individuals start showing periarticular bone loss in the absence of clinically evident synovitis (16).

We previously reported that ACPA-positive, treatment-naïve early RA patients already display reduced systemic bone mineral density (BMD) at presentation (17). Whether the negative impact of ACPA on bone continues over time after treatment institution remains largely unknown, and the few available studies have provided conflicting results (18, 19). In light of the multiplicity of factors influencing bone remodeling in RA, such as cumulative disease activity (20) and use of different medications with opposite effects on bone (21, 22), the net effect of ACPA, if any, could better emerge from prospective studies analyzing patient populations relatively homogeneous for disease duration and treatment. Here, we evaluated longitudinally the changes in BMD in relation to the autoantibody status in our inception cohort of early RA patients during the first two years of tightly controlled treatment according to standardized protocols.

## Patients and Methods

### Study Population and Treatment Protocol

We recruited 113 consecutive early RA patients from the Pavia Early Arthritis inception cohort (23, 24) after the institution in 2014 of standardized protocols for dual-energy x-ray absorptiometry (DXA) at both baseline and follow-up. Patients were treatment-naïve at inclusion, had symptoms of short duration (<12 months), and fulfilled the 2010 American College of Rheumatology (ACR)/European League Against Rheumatism criteria for RA (25). Patients with definitive diagnoses other than RA, or any suspicion of spondyloarthritis (including personal or familiar psoriasis and clinical or imaging enthesitis), were carefully excluded. After diagnosis, patients were started on a combination of low-dose prednisone (PDN) (5 mg/d) and methotrexate (MTX) from 15 mg/week, if not contraindicated. Alternative conventional synthetic (cs) disease modifying anti-rheumatic drugs (DMARDs) (leflunomide, sulfasalazine) were prescribed in patients with a contraindication (or early intolerance) to MTX; hydroxychloroquine was reserved to patients with very mild RA and/or severe comorbidities. Follow-up visits were scheduled every 2 months in the first semester, and then three-monthly, and treatment was adjusted to achieve low disease activity (LDA) according to the disease activity score on 28-joints (DAS28 <3.2). MTX was increased to up 25 mg/week. If the target of LDA had not been reached with the first csDMARD, a combination with another csDMARD or with a biologic (b) or targeted synthetic (ts) DMARD was considered based on the presence of poor prognostic factors (26). PDN was discontinued within the first year, when possible. Vitamin D supplements were prescribed to all patients and a calcium rich diet was encouraged. Bisphosphonates were prescribed according to international and national guidelines (27, 28) and to national reimbursement policies (http://www.aifa.gov.it/content/nota-79).

### Measurements

Demographic and general characteristics known to affect BMD were obtained by interview and are listed in **Table 1**. At baseline and regularly during follow-up, core variables of the DAS28 were recorded, including the tender and swollen joint count on 28 joints (TJC28, SJC28), the erythrocyte sedimentation rate (ESR) and C-reactive protein (CRP) levels. Rheumatoid factor (RF) and ACPA were centrally determined at baseline by immunonephelometry using a Dimension Vista 1500 system (Siemens, Erlangen, Germany) and by a second-generation Phadia ImmunoCAP 250 EliA CCP assay (Phadia, Freiburg, Germany). All patients underwent postero-anterior radiographs of the hands, wrists, and feet at baseline. Erosive RA was defined based on the presence of an erosion score ≥1 according to the Sharp/van der Heijde score (29). BMD measurements in the left hip and lumbar spine (LS), vertebrae L1-4, were performed at baseline and after 24 months using the same DXA equipment (Hologic, Waltham, Massachusetts, USA). All procedures were performed by two trained technicians in accordance with the manufacturer’s standardized procedures. BMD was expressed as absolute values (g/cm^2^) and Z-scores (in standard deviations [SDs] above or below the mean of a control population matched on age, sex and ethnicity) (30).

**Table 1:** Baseline demographic and clinical characteristics of the study population.

	Total	ACPA-pos	ACPA-neg	p
	n = 100	n = 43	n = 57	
**Demographics**				
Age, mean (SD), years	57.4 (13.5)	54.2 (11.8)	59.8 (14.3)	**0.04**
Female gender, n (%)	84 (84)	33 (76.7)	51 (89.5)	0.15
Post-menopausal, n (%)	54 (54)	18 (54.5)	36 (70.6)	0.20
Age at menopause, mean (SD), yrs	50.1 (4.2)	51.0 (3.3)	49.7 (4.5)	0.29
Premature menopause, n (%)	1 (1.9)	0 (0)	1 (2.8)	0.73
Caucasian, n (%)	98 (98)	42 (97.7)	56 (98.2)	0.59
BMI, mean (SD), kg/m^2^	25.7 (5.4)	24.3 (5.4)	26.7 (5.1)	**0.03**
Current smoker, n (%)	17 (17)	10 (23.3)	7 (12.3)	0.24
Alcohol ≥3 units/day, n (%)	2 (2)	1 (2.3)	1 (2.8)	0.63
Previous fracture, n (%)	3 (3)	1 (2.3)	2 (3.5)	0.81
Parent fractured hip, n (%)	8 (8)	3 (7)	5 (8.8)	0.97
Calcium supplementation, n (%)	2 (2)	0 (0)	2 (3.5)	0.61
Vitamin D supplementation, n (%)	3 (3)	1 (2.3)	2 (3.5)	0.81
Bisphosphonates use, n (%)	2 (2)	0 (0)	2 (3.5)	0.61
HRT, n (%)	2 (2)	0 (0)	2 (3.5)	0.61
**Disease characteristics**				
Symptom duration, median (IQR), weeks	21.4 (9.8-34.3)	21.4 (8.6-34.3)	21.4 (12.5-34.3)	0.62
1987 ACR criteria fulfilled, n (%)	83 (83)	37 (86)	46 (80.7)	0.67
DAS28, mean (SD)	4.17 (1.19)	3.77 (1.32)	4.47 (0.98)	**0.005**
SJC28, median (IQR)	4 (2-6)	3 (2-6)	4 (2.3-6)	0.25
TJC28, median (IQR)	6 (3-11)	3 (2-8)	8.5 (5-12)	**<0.001**
ESR, median (IQR), mm/1h	13.5 (5-29)	16 (6-30)	12 (5-27.3)	0.73
CRP, median (IQR), mg/dl	0.5 (0.3-1.2)	0.5 (0.3-1.1)	0.6 (0.3-1.2)	0.59
RF-positive, n (%)	41 (41)	34 (79.1)	7 (12.3)	**<0.001**
RF titer, median (IQR), U/ml	101 (42.5-234.5)	123 (46-232)	83 (29-236)	0.82
ACPA titer, median (IQR), U/ml	195 (38-340)			
Erosion SHS ≥1, n (%)	26 (26)	13 (30.2)	13 (22.8)	0.55
**DXA**				
***Spine L1-L4***				
BMD, mean (SD), g/cm^2^	0.936 (0.185)	0.934 (0.190)	0.937 (0.183)	0.95
Z score, mean (SD)	0.038 (1.540)	-0.271 (1.685)	0.347 (1.380)	**0.04**
Z score ≤-1, n (%)	26 (26)	14 (32.6)	12 (21.1)	0.29
***Femoral neck***				
BMD, mean (SD), g/cm^2^	0.726 (0.140)	0.724 (0.142)	0.728 (0.139)	0.88
Z score, mean (SD)	-0.073 (1.166)	-0.335 (1.209)	0.131 (1.100)	**0.04**
Z score ≤1, n (%)	22 (22)	14 (32.6)	8 (14)	0.05

ACPA, anti-citrullinated protein antibodies; ACR, American College of Rheumatology; BMD, bone mineral density; BMI, body mass index; CRP, C-reactive protein; DAS28, disease activity score on 28 joints; DXA, dual-energy X-ray absorptiometry; ESR, erythrocyte sedimentation rate; HRT, hormone replacement therapy; RF, rheumatoid factor; SD, standard deviation; SHS, Sharp van der Heijde score; SJC28, swollen joint count on 28 joints; TJC28, tender joint count on 28 joints.

Bold values indicate statistical significance with p < 0.05.

### Statistical Analysis

Data were described as mean and standard deviation (SD) or median and interquartile range (IQR) if continuous and as counts and percent if categorical. BMD variations at the LS and femoral neck (FN) after 24 months were expressed as absolute changes as well as percent changes from baseline, where negative values refer to overall bone loss. Comparisons between DXA values at baseline and 24 months were made through paired sample (each ACPA subgroup separately) and independent samples (ACPA-positive *vs* – negative) t-test. Predictors of BMD changes were analyzed by unadjusted and adjusted linear regression analysis. Variables with a p value ≤0.2 in univariate analysis were included. Age, gender, postmenopausal status and body mass index (BMI) were included in all multivariable models. Statistical analyses were performed using MedCalc^®^ Version 12.7.0.0 and the level of significance was set at 0.05.

## Results

### Baseline Characteristics of the Study Population

A total of 100 patients received BMD measurements at both the LS and FN at baseline and after 24 months, whilst 13 were lost to follow-up. Baseline demographic, clinical and densitometric characteristics of the 100 patients with complete data, also stratified for the ACPA status, are presented in **Table 1**.

ACPA-positive patients were significantly younger and with lower BMI compared to ACPA-negative patients. Other characteristics known to affect the BMD, such as smoking, alcohol intake, familiar osteoporosis, vitamin D supplements and use of bisphosphonates were comparable. The majority (>80%) of the patients in both ACPA subgroups also fulfilled the 1987 ACR classification criteria for RA (31). Median symptom duration at study inclusion was of approximatively 21 weeks in both ACPA-positive and –negative patients. Overall disease activity, as expressed by the DAS28, was significantly lower in ACPA-positive patients (mean difference [95% confidence interval, CI] 0.70 [0.22 to 0.18] points); however, such difference was mainly attributable to lower TJC28, whilst objective parameters of inflammation were comparable.

As expected (11, 14), ACPA-positive patients had significantly lower Z scores at both the spine (mean difference [95% CI] 0.618 [0.001 to 1.193]) and the FN (mean difference [95% CI] 0.466 [0.002 to 0.930]). After adjustment for age, gender, menopausal status and BMI, ACPA-positivity was associated with reduced BMD (Z score ≤-1 SD) with an odds ratio (OR) [95% CI] of 1.94 [0.73 to 5.12] at the spine and 2.80 [1.01 to 7.98] at the hip.

### Clinical Follow-Up

**Figure 1:**
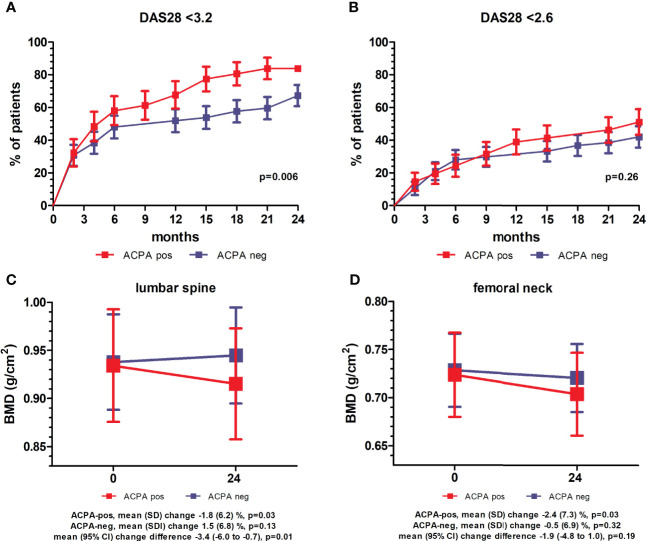
Clinical and densitometric follow-up stratified for anti-citrullinated protein antibodies. **(A, B)** Percentage of patients (± standard error) achieving low disease activity (LDA, DAS28 <3.2) **(A)** and remission (DAS28 <2.6) **(B)** over 24 months of follow-up stratified for anti-citrullinated protein antibodies (ACPA). p values refer to unadjusted Cox proportional-hazards regression. **(C, D)** Mean (95% confidence interval, CI) bone mineral density (BMD) at the lumbar spine **(C)** and femoral neck **(D)** at baseline and after 24 months in ACPA-positive and -negative patients. p values refer to pairwise comparisons between baseline and follow-up within ACPA-positive and –negative patients (paired samples t-test), and comparison of mean changes between ACPA-positive and –negative patients (independent samples t-test).

Collectively, after 24 months of treatment, LDA (DAS28 <3.2) was achieved by 78% of the patients, and remission (DAS28 <2.6) by 47%. Compared to ACPA-negative, ACPA-positive subjects more frequently achieved LDA, whilst the frequency of remission was similar (**Figure 1A, B**). Eighty-four percent of the patients was still on therapy with csDMARDs (MTX in 72.6% of the cases), whilst 13% had started a b/tsDMARD. Of the 81 patients prescribed with PDN at baseline, 51.9% was maintaining therapy at 24 months, with a mean (SD) cumulative dose of 2.8 (1.7) gr. Compared to ACPA-negative patients, ACPA-positive patients more frequently received a b/tsDMARD (25.6% *vs* 7%, p=0.02), whilst the mean (SD) PDN cumulative dose tended to be lower (2.5 [1.5] *vs* 3 [1.8] gr, p=0.16). Bisphosphonates were initiated in 36% of the patients (52.6% of ACPA-negative *vs* 32.6% of ACPA-positive, p=0.07), with a mean (SD) duration of treatment of 21.8 (10.4) months.

### Variations of BMD Over Follow-Up

At the 24 months assessment, the BMD at the LS had remained largely unchanged in the overall population (mean [SD] percent change 0.04 [6.7], p=0.78), whilst a small albeit significant decrease was observed at the FN (mean [SD] percent change -1.4 [7.1], p=0.02) (**Table 2**). BMD variations however clearly differed according to the autoantibody status (**Table 2** and **Figure 1C, D**). At the LS, the BMD in ACPA-positive patients declined significantly from 0.934 (0.190) to 0.915 (0.187) (p=0.03), corresponding to a decrease of 1.8% in 24 months. In contrast, in ACPA-negative patients, there was a trend for increased values from 0.932 (0.183) to 0.944 (0.181) (1.5%) (p=0.13). As such, none of the ACPA-positive patients with reduced BMD (Z score ≤-1) at baseline had returned to normal values as compared with 33.3% among ACPA-negative patients (p=0.07). In contrast, new BMD loss was observed in 10.3% of ACPA-positive and 6.7% of ACPA-negative patients with normal values at enrolment. At the FN, ACPA-positive patients again showed a significant reduction from 0.723 (0.142) to 0.704 (0.139) (p=0.03), corresponding to a decrease of 2.4%, whilst ACPA-negative patients were relatively stable. Also at the FN, none of the ACPA-positive patients regained normal BMD, and 6.9% additional patients underwent BMD reduction (Z score ≤-1) as compared with smaller variations in ACPA-negative subjects (**Table 2**). As a final result, the proportion of patients with reduced BMD (Z score ≤-1) after 24 months was significantly higher among ACPA-positive patients at both the spine (39.5% *vs* 19.3%, p=0.05) and the FN (37.2% *vs* 12.2%, p=0.007) (**Table 2**).

**Table 2:** Changes in BMD from baseline.

	Total	ACPA-pos	ACPA-neg	p
	n = 100	n = 43	n = 57	
**T24 DXA**				
***Spine L1-L4***				
BMD, mean change (SD), g/cm^2^	-0.002 (0.059)	-0.019 (0.056)*	0.012 (0.058)	**0.009**
BMD, mean % change (SD)	0.04 (6.74)	-1.84 (6.17)	1.54 (6.85)	**0.01**
Z score, mean change (SD)	0.128 (0.482)	-0.036 (0.488)	0.260 (0.439)*	**0.003**
Z score ≤-1, n (%)	28 (28)	17 (39.5)	11 (19.3)	0.05
Z score >-1 among patients with Z score ≤1 at baseline, n (%)	4/26 (15.4)	0/14 (0)	4/12 (33.3)	0.07
Z score ≤-1 among patients with Z score >-1 at baseline, n (%)	5/74 (6.8)	3/29 (10.3)	3/45 (6.7)	0.91
***Femoral neck***				
BMD, mean change (SD), g/cm^2^	-0.013 (0.056)*	-0.020 (0.056)*	-0.007 (0.055)	0.28
BMD, mean % change (SD)	-1.36 (7.14)	-2.43 (7.34)	-0.53 (6.94)	0.19
Z score, mean change (SD)	-0.012 (0.504)	-0.086 (0.460)	0.046 (0.534)	0.20
Z score ≤1, n (%)	23 (23)	16 (37.2)	7 (12.2)	**0.007**
Z score >-1 among patients with Z score ≤1 at baseline, n (%)	2/22 (9.1)	0/14 (0)	2/8 (25)	0.23
Z score ≤-1 among patients with Z score >-1 at baseline, n (%)	3/78 (3.8)	2/29 (6.9)	1/49 (2)	0.63

ACPA, anti-citrullinated protein antibodies; BMD, bone mineral density; DXA, dual-energy X-ray absorptiometry.

p values refer to inter-group comparisons between ACPA-positive and –negative patients (unpaired samples t-test).

*p < 0.05 for within-group comparisons between DXA at baseline and 24-months in the total cohort and in ACPA-positive and-negative patients as separate subgroups.

Bold values indicate statistical significance with p < 0.05.

At unadjusted linear regression, ACPA emerged significant predictors of larger reductions of the BMD at the LS, with a trend also at the FN (**Table 3**). No significant dose-dependent effects were observed for increasing ACPA levels, and no clear associations emerged for RF. Cumulative disease activity was not associated with increased bone loss. Of note, in patients achieving remission, BMD variations were not significantly different at neither the LS (mean [SD] -0.11 [7.67] *vs* 0.16 [5.92] p=0.84) nor the FN (mean [SD] -0.84 [7.49] *vs* -0.94 [6.86] p=0.53); similarly, time to achieve remission did not impacted on BMD loss (**Table 3**). Stratification according to the achievement of LDA provided similar results (not shown), irrespective of the time point at which LDA was achieved (**Table 3**). The use of bisphosphonates was instead highly protective. The cumulative PDN dose only impacted on variations at the LS. After adjusting for covariates, ACPA maintained independent association with more BMD loss at the LS but not the FN (**Table 3**). Confirming the independency from other variables, ACPA-positive patients receiving bisphosphonates still showed smaller increases in spine BMD compared to ACPA-negative patients (mean [SD] percent change 0.2 [4.5] *vs* 4.1 [6.2], p=0.11). Furthermore, restricting the analysis to subjects aged <65 years, BMD loss at the LS occurred only in ACPA-positive patients (mean [SD] percent change -2.7 [5.7] *vs* 0.7 [6.7], p=0.03).

**Table 3:** Predictors of BMD changes.

	% change of BMD lumbar spine		% change of BMD femoral neck	
Univariable analysis	r	p	r	p
Age	0.27 (0.07 to 0.44)	**0.008**	0.25 (0.06 to 0.43)	**0.01**
Female gender	0.09 (-0.12 to 0.28)	0.40	-0.12 (-0.31 to 0.08)	0.23
Menopause	0.14 (-0.08 to 0.35)	0.21	0.09 (-0.12 to 0.29)	0.41
BMI	0.04 (-0.16 to 0.24)	0.70	-0.11 (-0.30 to 0.09)	0.28
Smoking	-0.04 (-0.26 to 0.16)	0.67	0.01 (-0.20 to 0.21)	0.96
Achievement of LDA	-0.08 (-0.28 to 0.12)	0.41	0.03 (-0.17 to 0.23)	0.77
Achievement of remission	-0.02 (-0.22 to 0.18)	0.85	-0.06 (-0.26 to 0.14)	0.53
Time to LDA	0.08 (-0.12 to 0.28)	0.41	-0.06 (-0.25 to 0.14)	0.57
Time to remission	0.07 (-0.13 to 0.26)	0.52	-0.02 (-0.22 to 0.17)	0.81
Cumulative DAS28	0.02 (-0.18 to 0.22)	0.86	0.01 (-0.19 to 0.21)	0.91
ACPA	-0.25 (-0.43 to -0.05)	**0.01**	-0.13 (-0.32 to 0.07)	0.19
ACPA levels	0.00 (-0.20 to 0.20)	0.99	0.15 (-0.04 to 0.34)	0.12
RF	-0.11 (-0.31 to 0.09)	0.27	0.00 (-0.20 to 0.20)	0.99
RF levels	-0.07 (-0.27 to 0.13)	0.50	0.01 (-0.19 to 0.20)	0.94
Erosion SHS ≥1	-0.01 (-0.21 to 0.19)	0.91	0.10 (-0.10 to 0.30)	0.33
Cumulative PDN	-0.14 (-0.33 to 0.06)	0.16	-0.01 (-0.21 to 0.19)	0.93
Months of b/tsDMARDs	-0.16 (-0.35 to 0.04)	0.11	-0.06 (-0.26 to 0.14)	0.54
Months of bisphosphonates	0.32 (0.12 to 0.49)	**0.002**	0.26 (0.07 to 0.44)	**0.009**
**Multivariable analysis**	**β**	**p**	**β**	**p**
Age	–	–	–	–
Female gender	–	–	–	–
Menopause	–	–	–	–
ACPA	-2.33	**0.04**	–	–
Cumulative PDN	-0.00	**0.02**	NI	NI
Months of b/tsDMARDs	–	–	NI	NI
Months of bisphosphonates	0.16	**0.005**	0.14	**0.01**

Univariable and multivariable linear regression.

ACPA, anti-citrullinated protein antibodies; BMD, bone mineral density; b/ts, biological/targeted synthetic; BMI, body mass index; DAS28, disease activity score on 28 joints; DMARDs, disease modifying anti-rheumatic drugs; LDA, low disease activity; NI, not included; PDN, prednisone; RF, rheumatoid factor; SHS, Sharp van der Heijde score.

Bold values indicate statistical significance with p < 0.05.

## Discussion

Results from our analysis indicate that, despite suppression of inflammation and adoption of prophylactic measures, ACPA-positive early RA patients are exposed to increased risk of systemic bone loss, especially at the spine, in the first two years after treatment start. The small but significant decrease in hip BMD appears instead unexplained by disease and treatment-related variables.

The effects of early and intensive management on the epidemiology of OP in recent-onset RA remain poorly defined. Few studies have reported substantial stability of BMD in modern early RA populations (19, 32–35), with the highest rates of bone loss being observed at the hip in the first two years after treatment start (35), especially in patients receiving glucocorticoids (36). Although the exact rates of BMD variations are not comparable across studies due to different demographic characteristics, anti-rheumatic treatments and use of prophylactic measures, our results confirm that early RA patients collectively undergo only minor changes in systemic BMD upon tightly controlled management. Accordingly, a recent study found no significant difference in BMD at the spine and hip between individuals with RA in remission and those without RA (37). Our findings, however, do not contrast with the central role of inflammation in pathological bone remodeling. The tendency for more BMD loss at the LS in association with the use of b/tsDMARDs, together with the negative effects of higher cumulative PDN doses at this site, may indeed reflect the impact of a more refractory disease on bone. Our results also confirm the importance of prevention strategies for systemic OP (32, 33, 35). Indeed, treatment with bisphosphonates was the strongest predictor of maintenance of the BMD at any site. The reasons for the selective slight decline in the BMD at the FN irrespective of disease and treatment-related variables remain poorly understood, but recent studies have suggested that hip fragility may be an intrinsic characteristic of RA (38, 39).

Notwithstanding the overall reassuring effects of the modern management of RA on bone, systemic bone loss continues to progress in ACPA-positive patients early after treatment start, especially at the spine. The impact of ACPA beyond inflammation found here is in line with experimental (8–10) and clinical evidences (16, 17) and with recent studies demonstrating progression of bone erosions despite absent or minimal synovitis in ACPA-positive subjects (40, 41). Although RF also synergistically and dose-dependently affects the bone (11, 17), its effects may be missed in longitudinal studies, including our, in which fluctuations in autoantibody levels are not sequentially assessed. Our results however do not implicitly assign a prominent role to ACPA, and we are aware that a number of important RA-related and non-related factors, such as disease activity, type of medications and duration of follow-up might mitigate or even contrast the association between autoantibodies and early bone loss found here. Accordingly, Amkreutz et al. (19) recently failed to demonstrate significant relationships between ACPA and variations in BMD during five and ten years from treatment start in two independent populations of early RA and undifferentiated arthritis. However, also in this study, some possible differences seemed to arise, with the Swedish ACPA-positive sub-cohort showing a trend for reduced BMD at both the spine and the hip during the first two years, followed by further relative stabilization or even increase. This underscores the complexity in the longitudinal assessments of BMD variations in RA, where different factors may act in different directions and with different cumulative impact over time.

We acknowledge that, due to the relatively small sample size and some unbalances between ACPA-positive and -negative patients in certain variables affecting the bone, such as age and use of bisphosphonates, the independent effect of ACPA found in our study needs to be replicated. Age-related spondyloarthritis may have masked possible reductions in the BMD at the LS in ACPA-negative elderly patients. However, the relative stability of spine bone mass in seronegative subjects aged <65 yrs compared to seropositives corroborates the specific effect of ACPA. Equally important, the negative impact of RF on BMD at the spine despite systematic prophylaxis for OP has been already reported in early RA (35) and, also in our study, the protective effect of bisphosphonates on spine BMD was less evident in ACPA-positive patients.

In conclusion, ACPA positivity appears to impact on site-specific BMD, and remains an important predictor of bone density loss despite suppression of inflammation at least in the earliest phases of the disease. ACPA-positive patients should be therefore strictly monitored for the development of OP, and could benefit from anti-osteoporotic treatments irrespective of the presence of other risk factors.

## Data Availability Statement

The original contributions presented in the study are included in the article/supplementary material. Further inquiries can be directed to the corresponding author.

## Ethics Statement

The study was approved by the Local Ethical Committee of the IRCCS Policlinico San Matteo Foundation (n.08004598/b). The patients/participants provided their written informed consent to participate in this study.

## Author Contributions

SB conceived the work, contributed to the analysis and interpretation of data, and drafted the manuscript. LB contributed to the acquisition and interpretation of data and drafted the manuscript. AM conceived the work, contributed to the interpretation of data, and revised the manuscript critically for important intellectual content. LDS contributed to the acquisition of data and revised the manuscript critically. PD contributed to the acquisition of data and revised the manuscript critically. FM contributed to the acquisition of data and revised the manuscript critically. CM contributed to the interpretation of data and revised the manuscript critically for important intellectual content. All authors contributed to the article and approved the submitted version.

## Funding

This study was supported in part by funding from the IRCCS Policlinico San Matteo Foundation, Pavia, Italy.

## Conflict of Interest

The authors declare that the research was conducted in the absence of any commercial or financial relationships that could be construed as a potential conflict of interest.
